# Supplementary material for: Association of Four Dietary Patterns and Stair Climbing with Major Adverse Cardiovascular Events: A Large Population-Based Prospective Cohort Study
Source: Nutrients. 2024 Oct 22;16(21):3576. doi: 10.3390/nu16213576 (PMC11547348; doi:10.3390/nu16213576)
Supplement: Supplementary file 1 [file nutrients-16-03576-s001.zip › Table S1.pdf]

Table S1. Association between dietary patterns combined with stair climbing and MACE.

Dietary patterns and climbing stair	MACE			
	Case/N	HR (95% CI)	P value	P for interaction
**Alternate Mediterranean Diet score (AMED)**				<0.001
AMED T1 (0-3)				
Stair climbing (steps/day) None	271/2389	Reference		
Stair climbing (steps/day) 10-50	577/6312	0.97 (0.84 - 1.12)	0.702	
Stair climbing (steps/day) 60-100	970/11523	0.91 (0.79 - 1.04)	0.160	
Stair climbing(steps/day) 110-150	463/6012	0.86 (0.74 - 1)	0.050	
Stair climbing(steps/day) >150	358/4725	0.87 (0.74 - 1.02)	0.086	
AMED T2 (4-5)				
Stair climbing (steps/day) None	276/2598	0.9 (0.76 - 1.07)	0.229	
Stair climbing (steps/day) 10-50	545/6452	0.85 (0.74 - 0.99)	0.034	
Stair climbing (steps/day) 60-100	1052/13064	0.84 (0.74 - 0.97)	0.014	
Stair climbing(steps/day) 110-150	573/7168	0.86 (0.74 - 1)	0.044	
Stair climbing(steps/day) >150	443/5625	0.9 (0.77 - 1.05)	0.173	
AMED T3 (6-9)				
Stair climbing (steps/day) None	369/3758	0.87 (0.74 - 1.01)	0.073	
Stair climbing (steps/day) 10-50	668/8513	0.84 (0.73 - 0.96)	0.014	
Stair climbing (steps/day) 60-100	1370/19037	0.77 (0.68 - 0.88)	<0.001	
Stair climbing(steps/day) 110-150	789/10946	0.79 (0.69 - 0.91)	0.001	
Stair climbing(steps/day) >150	684/9262	0.85 (0.73 - 0.98)	0.023	
**Alternate Healthy Eating Index-2010 (AHEI-2010)**				<0.001
AHEI-2010 T1 (0-47)				
Stair climbing (steps/day) None	340/2847	Reference		
Stair climbing (steps/day) 10-50	700/7537	0.94 (0.82 - 1.07)	0.340	
Stair climbing (steps/day) 60-100	1251/14597	0.88 (0.78 - 0.99)	0.037	
Stair climbing(steps/day) 110-150	585/7574	0.81 (0.71 - 0.93)	0.002	
Stair climbing(steps/day) >150	476/5866	0.89 (0.78 - 1.03)	0.115	
AHEI-2010 T2 (48-58)				
Stair climbing (steps/day) None	292/2833	0.89 (0.76 - 1.05)	0.163	
Stair climbing (steps/day) 10-50	551/6863	0.86 (0.75 - 0.98)	0.027	
Stair climbing (steps/day) 60-100	1089/14275	0.83 (0.74 - 0.94)	0.003	
Stair climbing(steps/day) 110-150	645/7945	0.91 (0.8 - 1.04)	0.170	
Stair climbing(steps/day) >150	480/6318	0.88 (0.77 - 1.01)	0.074	
AHEI-2010 T3 (59-95)				
Stair climbing (steps/day) None	284/3065	0.9 (0.77 - 1.06)	0.205	
Stair climbing (steps/day) 10-50	539/6877	0.9 (0.78 - 1.03)	0.129	
Stair climbing (steps/day) 60-100	1052/14752	0.83 (0.74 - 0.94)	0.004	

Stair climbing(steps/day) 110-150	595/8607	0.83 (0.73 - 0.95)	0.007	
Stair climbing(steps/day) >150	529/7428	0.9 (0.78 - 1.03)	0.121	
**Dietary Approaches to Stop Hypertension (DASH)**				<0.001
DASH T1 (0-20)				
Stair climbing (steps/day) None	320/2743	Reference		
Stair climbing (steps/day) 10-50	661/7429	0.94 (0.82 - 1.07)	0.347	
Stair climbing (steps/day) 60-100	1142/13976	0.87 (0.77 - 0.98)	0.027	
Stair climbing(steps/day) 110-150	581/7239	0.89 (0.78 - 1.02)	0.099	
Stair climbing(steps/day) >150	444/5501	0.93 (0.81 - 1.08)	0.329	
DASH T2 (21-25)				
Stair climbing (steps/day) None	284/2834	0.86 (0.74 - 1.02)	0.076	
Stair climbing (steps/day) 10-50	620/7025	0.91 (0.8 - 1.05)	0.189	
Stair climbing (steps/day) 60-100	1192/14488	0.87 (0.77 - 0.99)	0.032	
Stair climbing(steps/day) 110-150	609/8050	0.82 (0.72 - 0.94)	0.005	
Stair climbing(steps/day) >150	507/6424	0.9 (0.78 - 1.04)	0.145	
DASH T3 (26-40)				
Stair climbing (steps/day) None	312/3168	0.92 (0.79 - 1.08)	0.311	
Stair climbing (steps/day) 10-50	509/6823	0.83 (0.72 - 0.96)	0.010	
Stair climbing (steps/day) 60-100	1058/15160	0.79 (0.7 - 0.9)	0.000	
Stair climbing(steps/day) 110-150	635/8837	0.83 (0.72 - 0.95)	0.007	
Stair climbing(steps/day) >150	534/7687	0.84 (0.73 - 0.96)	0.013	
**Healthful Plant-based Diet Index (HPDI)**				<0.001
HPDI T1 (0-53)				
Stair climbing (steps/day) None	301/2705	Reference		
Stair climbing (steps/day) 10-50	653/7156	1.01 (0.88 - 1.16)	0.867	
Stair climbing (steps/day) 60-100	1137/13467	0.94 (0.83 - 1.07)	0.336	
Stair climbing(steps/day) 110-150	546/6889	0.91 (0.79 - 1.05)	0.209	
Stair climbing(steps/day) >150	459/5323	1.04 (0.9 - 1.2)	0.598	
HPDI T2 (54-60)				
Stair climbing (steps/day) None	338/3025	1.05 (0.9 - 1.23)	0.538	
Stair climbing (steps/day) 10-50	635/7450	0.95 (0.83 - 1.09)	0.500	
Stair climbing (steps/day) 60-100	1242/15551	0.92 (0.81 - 1.05)	0.215	
Stair climbing(steps/day) 110-150	645/8563	0.89 (0.77 - 1.02)	0.091	
Stair climbing(steps/day) >150	506/6851	0.9 (0.78 - 1.04)	0.159	
HPDI T3 (61-62)				
Stair climbing (steps/day) None	277/3015	0.94 (0.8 - 1.11)	0.453	
Stair climbing (steps/day) 10-50	502/6671	0.92 (0.8 - 1.06)	0.250	
Stair climbing (steps/day) 60-100	1013/14606	0.86 (0.75 - 0.98)	0.023	
Stair climbing(steps/day) 110-150	634/8674	0.93 (0.81 - 1.07)	0.297	
Stair climbing(steps/day) >150	520/7438	0.93 (0.81 - 1.08)	0.337	

Note: Model was adjusted for age, sex, race/ethnicity, education, Townsend deprivation index, drinking status, smoking status, total energy intake, physical activity, sleep duration, BMI, systolic blood pressure, diastolic blood

pressure, diabetes, hypertension, hypercholesterolemia, obesity (BMI >30kg/m^2), atherosclerotic disease, and history of cardiovascular disease. Abbreviations: MACE, major adverse cardiovascular events.
