# Supplementary figures and images for: Association of Four Dietary Patterns and Stair Climbing with Major Adverse Cardiovascular Events: A Large Population-Based Prospective Cohort Study
Source: Nutrients. 2024 Oct 22;16(21):3576. doi: 10.3390/nu16213576 (PMC11547348; doi:10.3390/nu16213576)

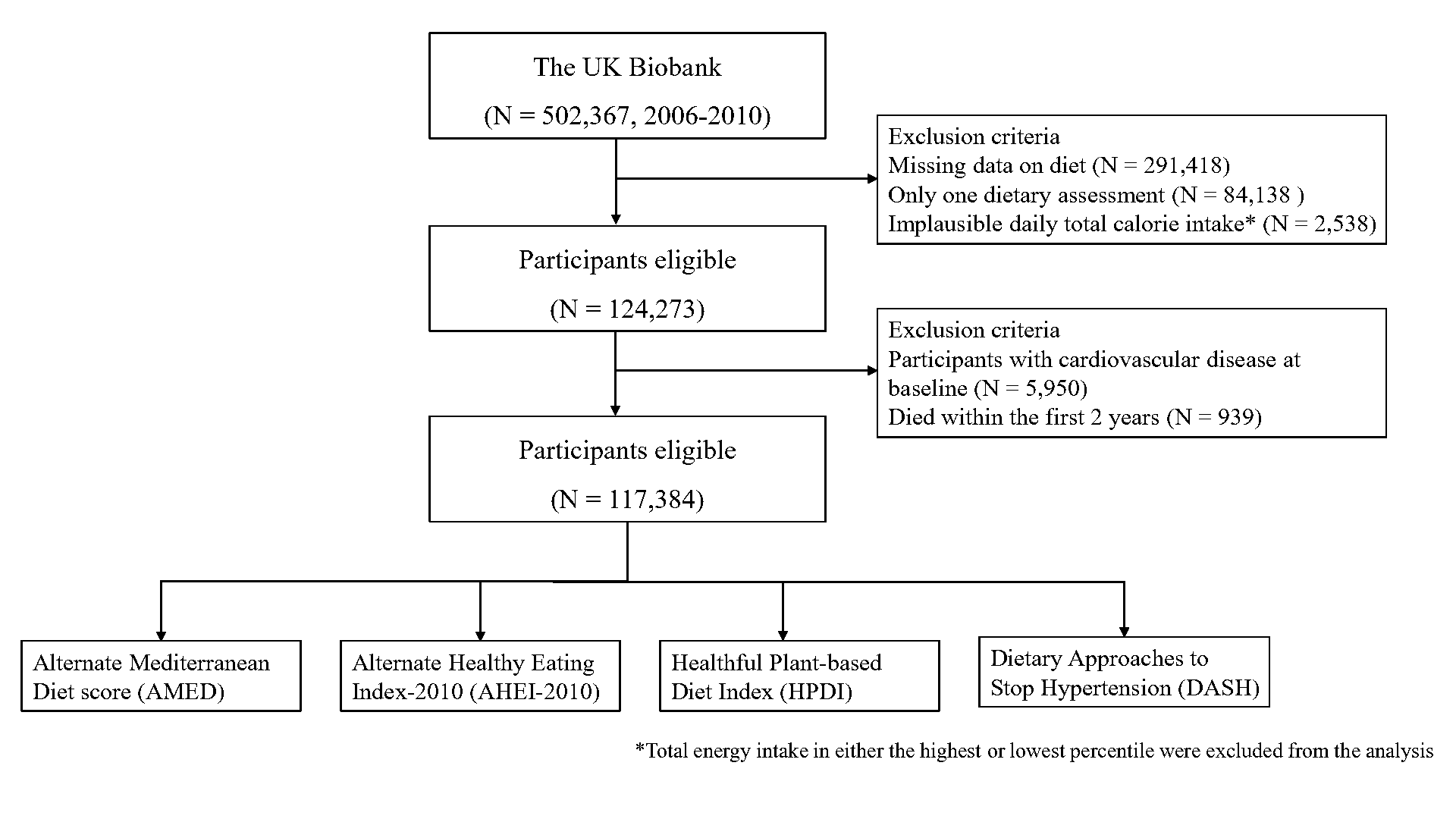

Supplement: Supplementary file 1 [file nutrients-16-03576-s001.zip › Figure S1.tif]
